# Numerical simulation of ISFET structures for biosensing devices with TCAD tools

**DOI:** 10.1186/1475-925X-14-S2-S3

**Published:** 2015-08-13

**Authors:** Daniele Passeri, Arianna Morozzi, Keida Kanxheri, Andrea Scorzoni

**Affiliations:** 1 Dipartimento di Ingegneria, Università degli Studi di Perugia, via G. Duranti 93, Perugia, Italy; 2 Dipartimento di Fisica e Geologia, Università degli Studi di Perugia, via Pascoli 1, Perugia, Italy

## Abstract

**Background:**

Ion Sensitive Field Effect Transistors (ISFETs) are one of the primitive structures for the fabrication of biosensors (BioFETs). Aiming at the optimization of the design and fabrication processes of BioFETs, the correlation between technological parameters and device electrical response can be obtained by means of an electrical device-level simulation. In this work we present a numerical simulation approach to the study of ISFET structures for bio-sensing devices (BioFET) using Synopsys Sentaurus Technology Computer-Aided Design (TCAD) tools.

**Methods:**

The properties of a custom-defined material were modified in order to reproduce the electrolyte behavior. In particular, the parameters of an intrinsic semiconductor material have been set in order to reproduce an electrolyte solution.

By replacing the electrolyte solution with an intrinsic semiconductor, the electrostatic solution of the electrolyte region can therefore be calculated by solving the semiconductor equation within this region.

**Results:**

The electrostatic behaviour (transfer characteristics) of a general BioFET structure has been simulated when the captured target number increases from 1 to 10. The *I_D _*current as a function of the *V_DS _*voltage for different positions of a single charged block and for different values of the reference electrode have been calculated.

The electrical potential distribution along the electrolyte-insulator-semiconductor structure has been evaluated for different molar concentrations of the electrolyte solution.

**Conclusions:**

We presented a numerical simulation approach to the study of Ion-Sensitive Field Effect Transistor (ISFET) structures for biosensing devices (BioFETs) using the Synopsys Sentaurus Technology Computer-Aided Design (TCAD) tools.

A powerful framework for the design and optimization of biosensor has been devised, thus helping in reducing technology development time and cost. The main finding of the analysis of a general reference BioFET shows that there is no linear relationship between the number of charges and the current modulation. Actually, there is a strong position dependent effect: targets localized near the source region are most effective with respect to targets localized near the drain region. In general, even randomly distributed targets are more efficient with respect to locally grouped targets on the current modulation. Moreover, for the device at hand, a small positive biasing of the electrolyte solution, providing that the transistor goes on, will result in a greater enhancement of the current levels, still retaining a good sensitivity but greatly simplifying the operations of a real device.

## Background

The integration of biologically active materials, such as molecules (enzymes, antibodies, antigens, proteins or nucleic acids) and/or biological systems (cells, plants, tissues, organs) with Ion Sensitive Field Effect Transistors (ISFETs) is one of the key elements for the fabrication of the class of biosensors referred to as BioFETs. The aim is to build an hybrid functional system, able to couple the unique (bio)receptor system capabilities with an electrical read-out and acquisition system. Silicon Field Effect Transistors (FETs) are nowadays the primitive element of the new generation of biosensors, since BioFETs can be built from the basic ISFET structure by modifying the gate of the transistor or by coupling the gate oxide with biological sensing elements (receptors).

Aiming at the optimization of the design and fabrication processes of BioFETs, the correlation between technological parameters and device electrical response should more directly be obtained by means of an electrical device-level simulation. To this purpose, different approaches have been proposed in literature [1-3] both at device and circuit level [4-6]. In particular, in the approach proposed in [6] the incorporation of a physical model of the electrolyte-insulator-semiconductor (EIS) structure into a numerical device simulator has been carried out. The EIS system equations are coupled with the charge-transport equations and solved self-consistently on the discretized domain, thus resulting in a "custom" simulation tool.

In this work, we rely on the state-of-the-art commercial Synopsys Sentaurus TCAD packages. Sentaurus is a suite of TCAD tools which simulates the fabrication, operation and reliability of semiconductor devices [7]. The Sentaurus simulators use physical models to represent the device fabrication steps and operation, thereby fostering the exploration and optimization of new semiconductor devices. The adoption of TCAD tools reduces technology development time and cost at the same time providing insight into advanced physical phenomena through self-consistent multidimensional modelling capabilities, improving device design, yield, and reliability. However, the direct device level simulation of an electrolyte solution in Sentaurus TCAD is not straightforward: actually, the suite of standard materials does not include any electrolyte.

## Methods

The properties of a custom-defined material were therefore modified in order to reproduce the electrolyte behavior. In particular, the parameters of an intrinsic semiconductor material have been set in order to reproduce an electrolyte solution: the permittivity of the material can be set as simulation input parameter, depending on the type of electrolyte, thus reproducing the real, measured, conductivity of the solution. In this case the permittivity and the refractive index were set in order to reproduce the behavior of water. The bandgap energy dependence on the temperature is modelled as

(1) $$E_{g}\left(T\right)=E_{g}\left(0\right)-\frac{\alpha T^{2}}{T+\beta }$$

where *α *and *β *are material dependent parameters and *Eg*(0) is the bandgap energy at *T *= 0K. We set the *Eg*(0) = 1.5eV thus satisfying the requirement$\left(E_{g}/2-q\varphi \right)\gg kT$, i.e. greater than a few thermal energies (*q *is the elementary charge and *φ *is the electrical potential of the material). With this approximation the Poisson-Boltzmann (PB) equation, describing the charge distribution in the electric double layer, can be viewed as the semiconductor equation applied to an intrinsic material [8]. By replacing the electrolyte solution with an intrinsic semiconductor, the electrostatic solution of the electrolyte region can therefore be calculated by solving the semiconductor equations within this region.

The Shockley-Read-Hall (SRH) statistics has been adopted for the generation/recombination processes modelling, by setting the maximum recombination time according to literature findings [9]. In order to account for the surface effects on the carrier mobility, the simplified Lombardi model was used [10]. Actually, in the channel region of a FET, the high transverse electric field forces carriers to interact strongly with the semiconductor-insulator interface. Carriers are subjected to scattering by acoustic surface phonons and surface roughness. This model can describe the mobility degradation caused by these effects; the maximum mobility values have been set to $\mu_{p}^{max}=4.98\cdot 10^{-4}\text{c}{\text{m}}^{2}/\text{V}\cdot \text{s}$ and to$\mu_{n}^{max}=6.88\cdot 10^{-4}\text{c}{\text{m}}^{2}/\text{V}\cdot \text{s}$ respectively, to reproduce the behavior of *Na^+ ^*and *Cl^- ^*ions in a *NaCl *solution [16]. Actually, the maximum mobility values of ionic species can be freely set as well as simulation input parameters. It should be noticed that the carrier mobility is much lower with respect to standard free carrier mobility of an intrinsic semiconductor, thus consistently miming the behavior of ions in a real ionic solution.

Eventually, different ion concentrations within the solution are correlated to the free carriers within the equivalent semiconductor through the densities of states which can be set as input parameters, according to the pH of the solution.

The semiconductor state densities within the conduction and the valence bands, *N_C _*and *N_V_*, are the most significant parameters that correlate the physical properties of an electrolyte solution to the electrical parameters of an intrinsic semiconductor. Within this framework, the electrons and holes represent the mobile ions in the solution. The density of states *N_C _*and *N_V _*were therefore specified according to the molar concentration of the ionic solution, according to the following procedure. If we consider the *H*_2_*O *dissociation $H_{2}O+H_{2}O\to H_{3}O^{+}+OH^{-}$ at the chemical equilibrium, the concentration of [*OH*^-^] and [*H*_3_*O*^+^] are correlated by the ionic product for water

(2) $$K_{W}=\left[H_{3}O^{+}\right]\left[OH^{-}\right]$$

This value is strongly dependent on the temperature; however, at T = 25 °C it reads *K_W _*= 10^-14 ^[11]. The analogy with the electrons and holes concentrations in a semiconductor can be accomplished by accounting for the *mass action law*, stating that under thermal equilibrium the product of the free electron concentration *n *and the free hole concentration *p *is equal to a constant equal to the square of intrinsic carrier concentration. If the number of carriers is much less than the number of band states, the carrier concentrations can be approximated by using the Boltzmann statistics, giving

(3) $$n≅N_{C}e^{-\frac{E_{C}-E_{f}}{kT}}$$

and

(4) $$p≅N_{V}e^{-\frac{E_{f}-E_{V}}{kT}}$$

where *E_C _*is the lower energy limit of the conduction band, *E_V _*is the upper energy limit of the valence band, *E_f _*is the Fermi level and *k *is the Boltzmann constant. By combining (3) and (4) if *n *= *p*

(5) $$np=N_{C}N_{V}e^{-\frac{E_{G}}{kT}}=n_{i}^{2}$$

The effective density of states for electrons in the conduction band and for holes in the valence band are calculated from $N_{C}=2{\left[\frac{2\pi m_{e}^{\text{*}}kT}{h^{2}}\right]}^{3/2}$ and $N_{V}=2{\left[\frac{2\pi m_{h}^{\text{*}}kT}{h^{2}}\right]}^{3/2}$ where $m_{e}^{*}$ and $m_{h}^{*}$ are the effective mass of electrons and holes for density of states calculations, and *h *is the Planck constant.

For the calculation of the electrolyte "equivalent" semiconductor *N_C _*and *N_V _*values, we can consider the Avogadro constant $N_{A}=6.022\cdot 10^{23}\text{mo}{\text{l}}^{-1}$ to convert $1\text{mol}/\text{L}\to 6.022\cdot 10^{23}\left[\;\right]/\text{L}\to 6.022\cdot 10^{20}\left[\;\right]/\text{c}{\text{m}}^{3}$ and assuming $\left[H_{3}O^{+}\right]\equiv p,\left[OH^{-}\right]\equiv n$. Therefore, for instance, for a solution with *pH *= 7 the concentration of the ion $\left[H_{3}O^{+}\right]=10^{-7}\text{mol}/\text{L}$ corresponds to the hole concentration $p=10^{-7}\times 6.022\cdot 10^{20}\left[ions\right]/\text{c}{\text{m}}^{3}=6.022\cdot 10^{13}\left[ions\right]/\text{c}{\text{m}}^{3}$. By substituting this value in the expression (4), the "equivalent" density of states in the valence band can be calculated, by considering, as previously introduced, the energy gap *E_g _*= 1.5eV and obtaining

(6) $$N_{V}=2.4\times 10^{26}\text{c}{\text{m}}^{-3}.$$

A similar procedure can be used to determine the density of states in the conduction band. For a *pH *= 7 the concentration of both ion species are the same, i.e. $\left[H_{3}O^{+}\right]=\left[OH^{-}\right]$ therefore we calculate$n=10^{-7}\times 6.022\cdot 10^{20}\left[ions\right]/\text{c}{\text{m}}^{3}=6.022\cdot 10^{13}\left[ions\right]/\text{c}{\text{m}}^{3}$ corresponding to

(7) $$N_{C}=2.4\times 10^{26}\text{c}{\text{m}}^{-3}.$$

The relations (6) and (7) hold for a *pH *= 7 solution. However, this is not a limiting case. By considering the ionic product of water, the ionic species concentrations can be translated to carrier concentrations, depending on the concentration of the solution. In other words, for any given *pH *value of an equilibrium state solution at a constant temperature *T*, it is possible to determine the concentration of *n *and *p *of the equivalent intrinsic semiconductor, and therefore the values of *N_C _*and *N_V_*.

## Results

### ISFET simulation

**Figure 1:**
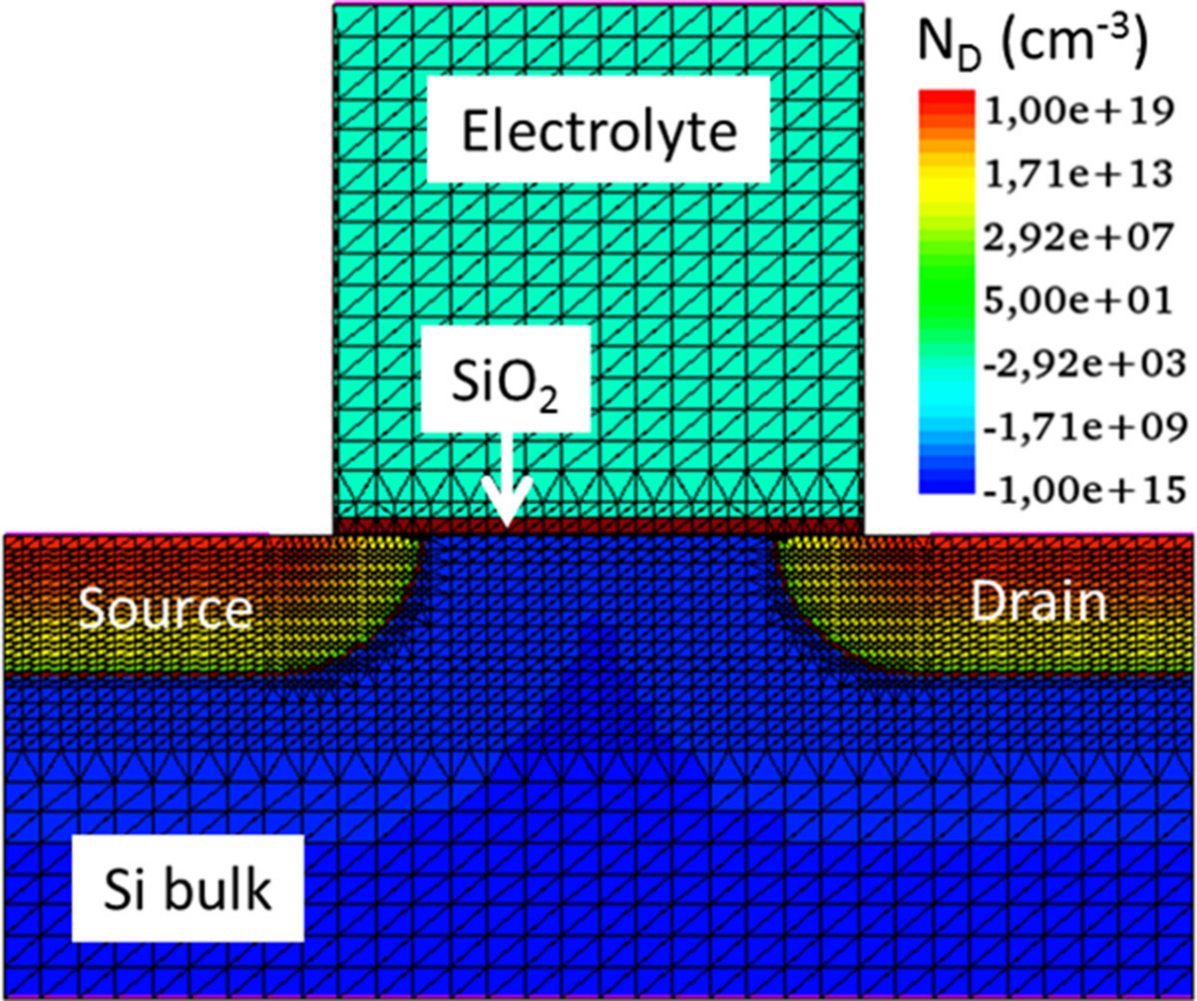
**ISFET sketch**. Cross-section of the simulated ISFET device

**Figure 2:**
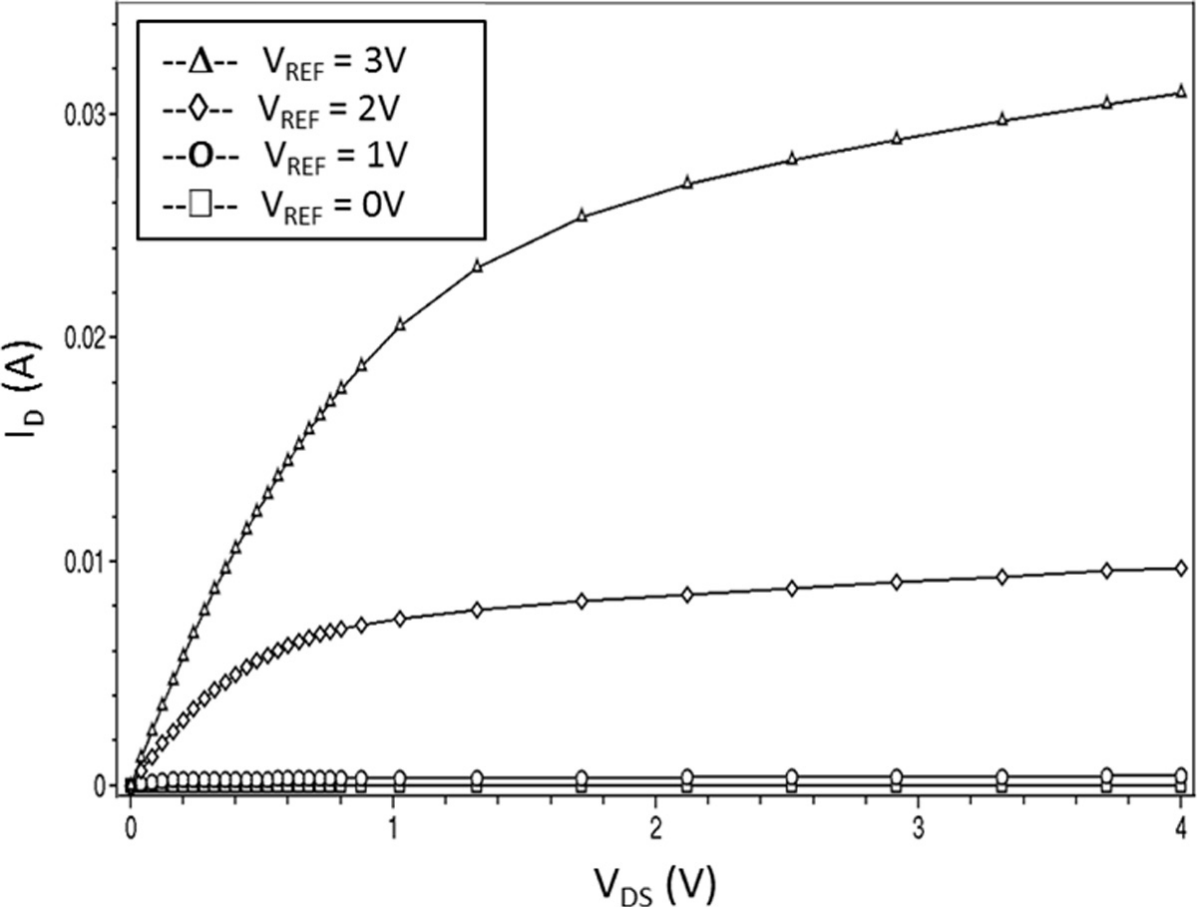
**ISFET simulation**. Output characteristics (I_D _vs. V_DS_) of the ISFET device.

In order to check the suitability of the modelling procedure, an ISFET device has been simulated. The 2D cross-section of the simulated structure is reported in Figure 1, along with its discretization mesh. The static characteristic of the device, namely the *I_D _*- *V_DS _*curves for different biasing voltages of the reference electrode are reported in Figure 2. The qualitative behaviour of the devices is very similar to the classical MOSFET *I_D _*- *V_DS _*curves, as expected.

**Figure 3:**
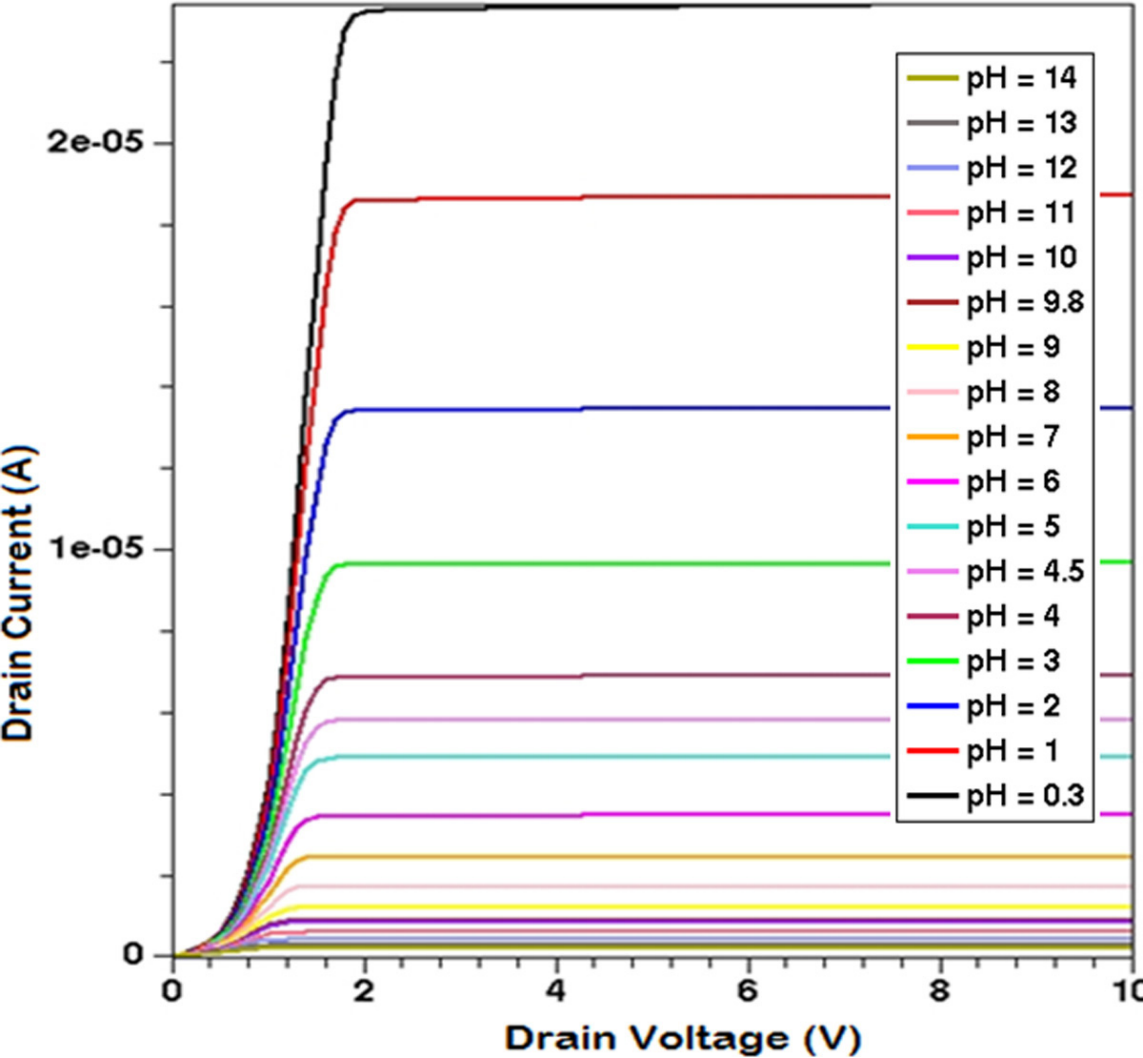
**ISFET as a pH sensor - 1**. Drain current as a function of the drain voltage at different pH solution concentrations (V_REF _= 0).

**Figure 4:**
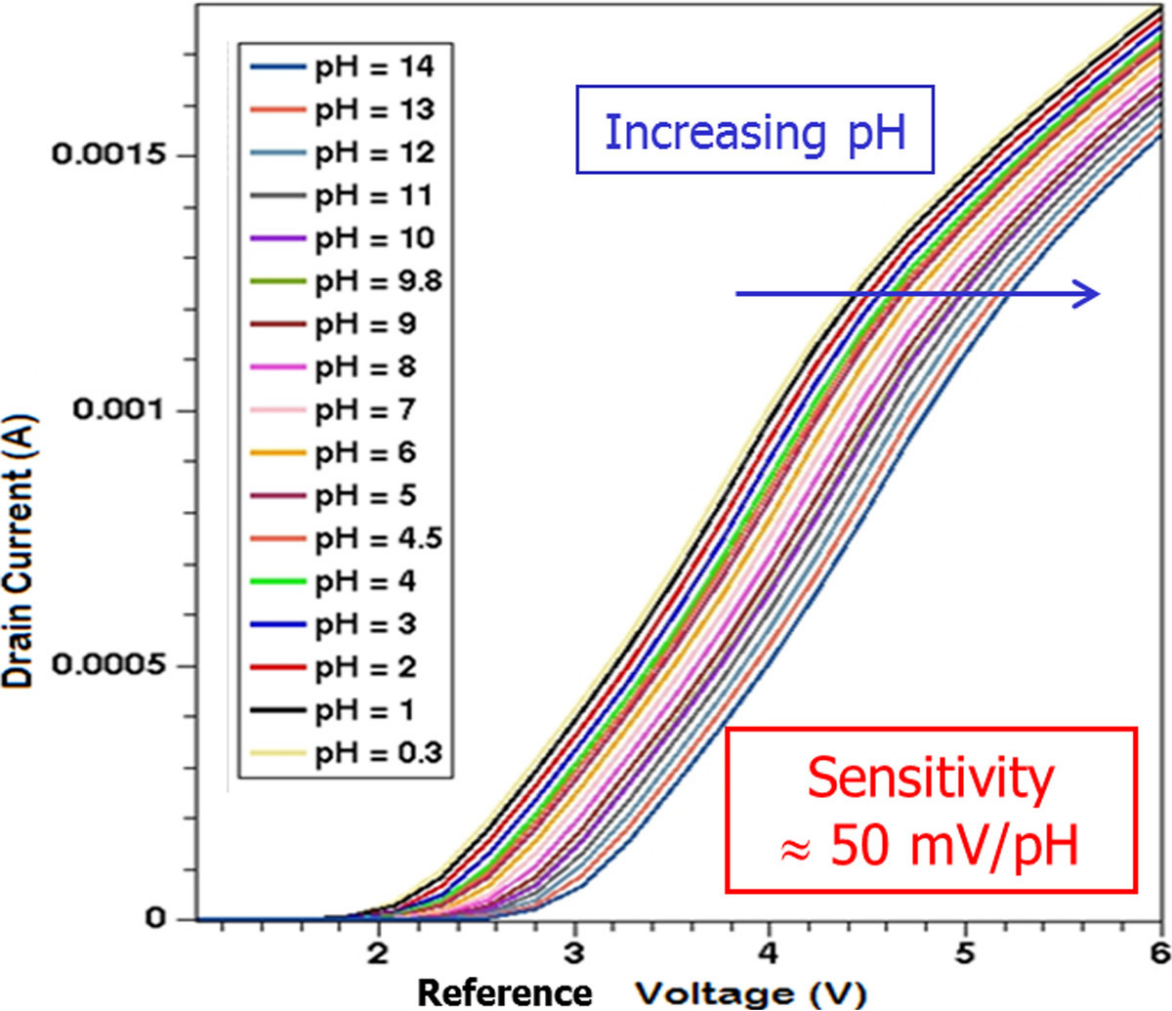
**ISFET as a pH sensor - 2**. Drain current as a function of the reference voltage at different pH solution concentrations (V_DS _= 2 V).

By varying the solution *pH*, e.g. the concentration of ionic species, the sensitivity of the ISFET device as *pH *sensor can be evaluated (Figure 3 and Figure 4). By considering a reference value of *V_DS _*= 2V, the family of curves of the drain current as a function of the reference electrode voltage can be calculated, by varying the densities of states of the electrolyte material according to the solution *pH*. A sensitivity of about 50mV/*pH *in terms of threshold voltage shift has been found, in agreement with literature data ([6], [9]).

### Device simulation of a general BioFET device

Once assessed the suitability of the methodology, a physically sound modelling scheme of BioFET sensors has been set-up. The label-free electrical biosensors rely on the field effect induced by charges of target biomolecules in an electrolyte environment. In real devices, receptor probes are immobilized on the surface of an electrolyte-insulator-semiconductor system so that the target molecules are bound to the probes by the bio-affinity phenomenon. The localized, fixed charges induce the field effect on the underlying conduction channel that leads to the current modulation. The channel modulation effect induced by irregular charge distribution can scarcely be estimated through analytical methods. Moreover, significant effect have been observed at very low target concentration when only a small portion of the receptor probes is bound to the target molecules. On the other hand, the detailed analysis of the effect of the actual charge distributions could conveniently be obtained through an accurate numerical simulation method [8]. The proposed methodology guarantees the self-consistent modelling of very different types of material regions, such as semiconductor, electrolyte solution and organic molecule regions. In particular, a realistic picture of the charge distribution can be obtained as a cluster of charges on the electrolyte region due to target molecules which are bound randomly to a receptor site. When a binding reaction occurs at a certain position on the surface, a given charge density is localized in that specific position.

In this work, we propose this methodology to devise a BioFET aimed at the study of electrophysiological neuronal activity. It has been already pointed out in the past that ISFET devices can measure the extracellular voltage of a single neuron attached with its cell membrane to the device insulator in an open gate configuration [12-14]. The change of the extracellular voltage induced by the neuron gives rise to an electric field across the insulator that modulates the drain-to-source current of the ISFET [15].

**Figure 5:**
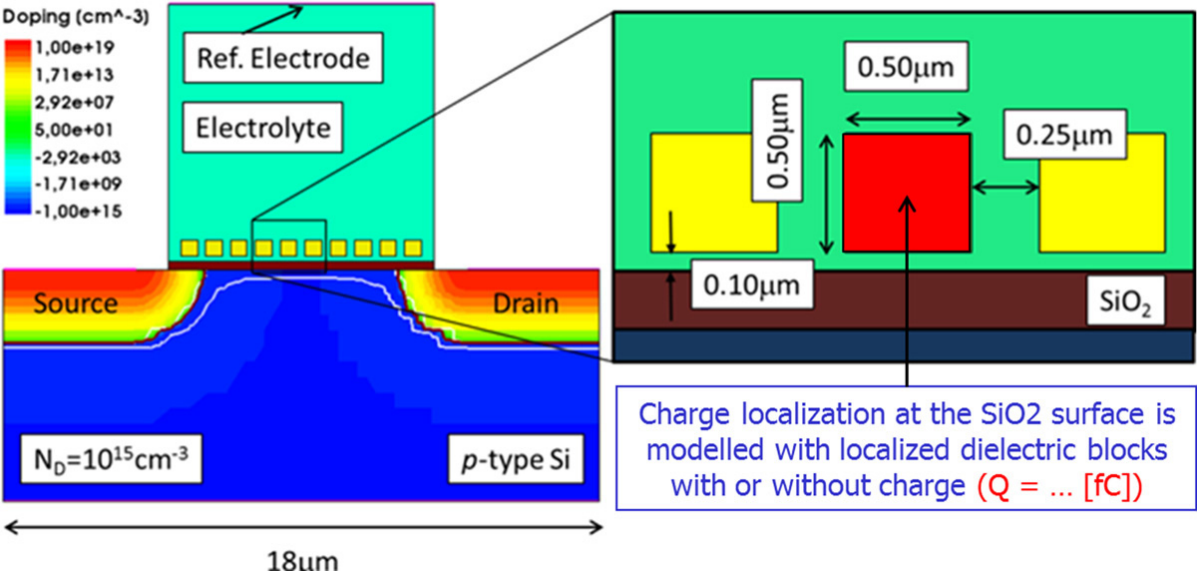
**BioFET simulated structure**. Sketch of the simulated structure: charge localization at the SiO_2 _surface is modelled with localized dielectric block with or without charge.

A sample simulated structure is therefore shown in Figure 5. The whole system is based on a standard ISFET device, featuring a *p*-type low doped Si substrate, a thin SiO_2 _interface and an electrolyte solution with a top reference electrode. The effect of spatially localized charges due to immobilization of target molecules is reproduced by means of a number of small blocks of dielectric material whose dimensions are compatible with the dimension of the target cells (we assume that the shape of the cell does not affect the charge distribution). The dimensions of the blocks and their distance from the dielectric surface can be chosen as design parameters, in order to account for different kinds of biosensors (e.g. target specificity and/or electrolyte characteristics which define the receptor size and position). For the device at hand, a proper "segmentation" of the gate of the ISFET structure previously simulated has been obtained by considering ten receptors with 0.5 *μm *length (Figure 5).

When the affinity reaction occurs at the receptor site (i.e., receptor and target molecule bind creation), a given charge distribution is assigned to each single block. The charge state of each block is assumed to be neutral (*Q *= 0) before the reaction occurs, even if it is possible to model their actual value (being positive or negative), as a reference state to be compared with the situation when a binding reaction occurs and a charge is localized (*Q *= *Q_T_*). In this case, a value of $|Q_{T}|=4.8\cdot 10^{-16}C$ has been used. The value (order of magnitude) of the fixed charge has been taken from literature [8] as a reference value of the charge localized when a Streptavidin molecule is sensed on a sensor surface. Due to the *p*-type Si substrate doping concentration, the opposite polarity of the fixed charge is found to be the most effective on the modulation of the FET electrical behaviour. However, both signs of the localized charge (positive or negative) can be taken into account, representing different localized molecules (e.g. Avidin or Streptavidin ).

The aim is to evaluate the effect of the same amount of charge expected in Silicon NanoWire (SiNW) FET biosensors over the electric potential distribution of the proposed structure (which is much larger in terms of dimensions and distances).

**Figure 6:**
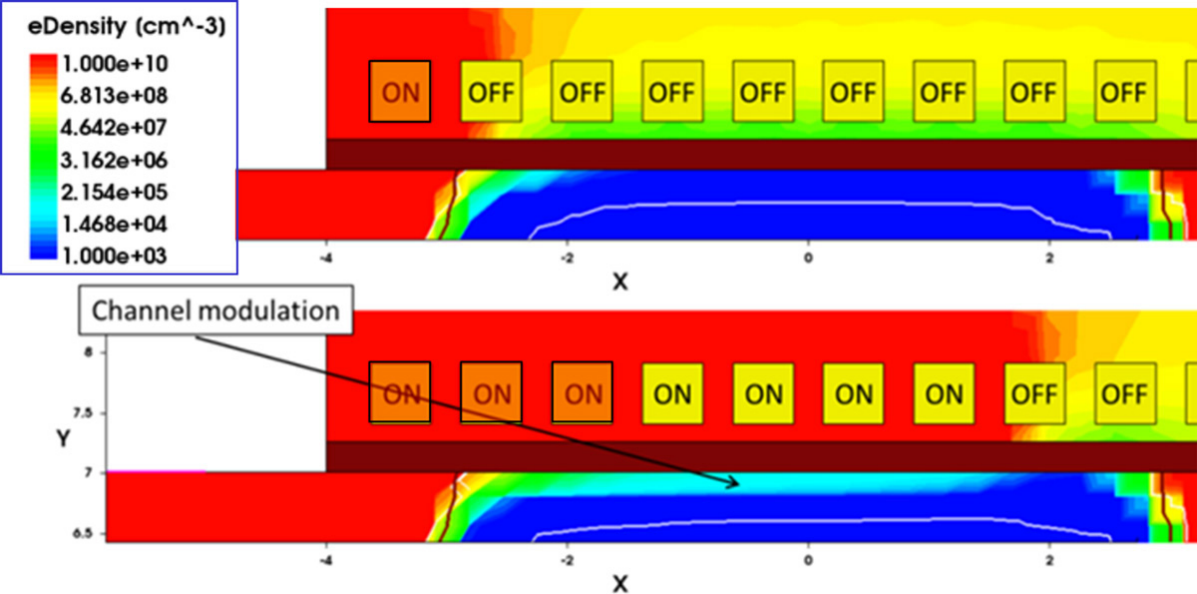
**BioFET channel modulation**. Channel modulation as a function of the number of the occupied receptors: electron density along the channel.

The electrostatic behaviour (transfer characteristics) of the structure has been simulated when the captured target number increases from 1 to 10. In a random distributed charge modelling, however, the static characteristics cannot be represented by a single curve. Actually, the transistor channel can have different conductance values depending on the bound target positions despite the fact that the number of bound targets is the same. In particular, the electron density along the channel depends strongly on the number and position of charged receptors (Figure 6). Since the conductance does not have a linear relationship with the target charge, the overall conductance modulation cannot be obtained through a linear combination of the modulation effects induced by each target. Therefore, one should simulate every case of the receptor-target binding combination to obtain a complete set of conductance results. This is of course an overwhelming computational effort; we therefore simulated a still huge number of several randomly selected sample cases.

**Figure 7:**
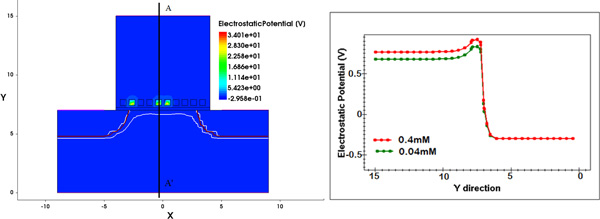
**BioFET electrical potential**. Electrostatic potential along a vertical structure cut (on the right) at different solution concentrations.

However, thanks to the TCAD simulation environment set-up, the microscopic electrical quantities can be analysed as a function of the BioFET technological parameters and/or the electrolyte characteristics. As an example, the electrical potential distribution along the electrolyte-insulator-semiconductor structure can be evaluated for different molar concentrations of the electrolyte solution (Figure 7). This is important in order to deeply evaluate the variation of microscopic quantities (e.g. the electric potential distributions, as well as electron and hole concentrations) as a function of the external conditions; this will eventually result in macroscopic quantities changes (i.e. calculated current at the output electrodes) which can be better understood.

**Figure 8:**
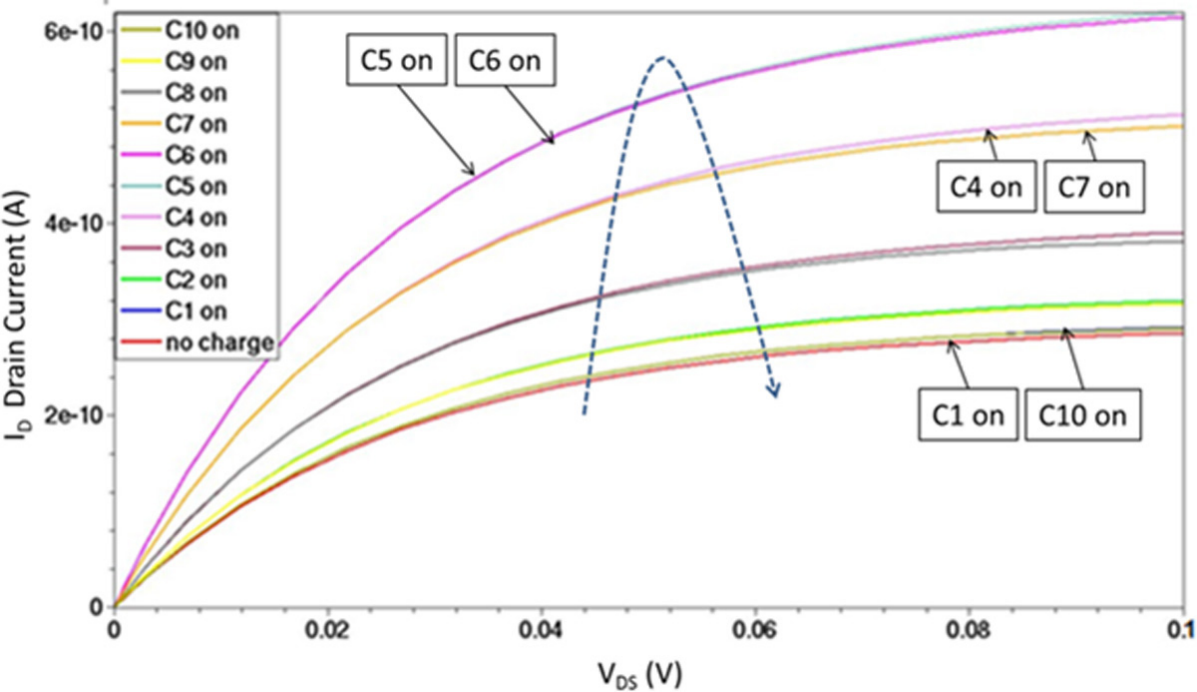
**BioFET drain current**. Drain current as a function of the reference voltage, depending on the position of a single charged receptor.

A summary plot is reported in Figure 8 where is shown the *I_D _*current as a function of the *V_DS _*voltage for different positions of a single charged block. The reference electrode voltage was set to ground, e.g. *V_REF _*= 0V. Starting from the lower curve (when no localized charge at all is experienced, i.e. when no reaction has occurred), the current tends to increase with the position of the charged block moving from the source channel region (C1 *on*) toward the middle between source and drain (C5 *on *or C6 *on*), and eventually decreasing when the charge approaches the drain region (C10 *on*).

**Figure 9:**
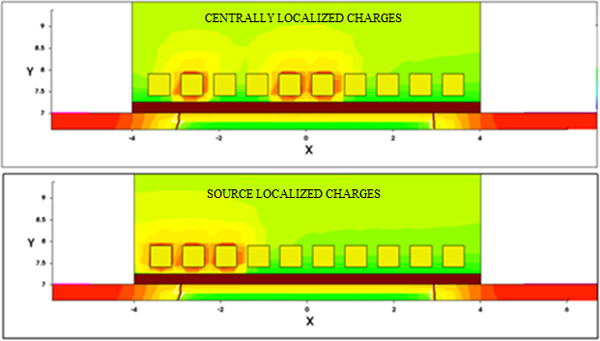
**BioFET channel charge distribution**. Channel charge density: localized vs. distributed charged receptors.

**Figure 10:**
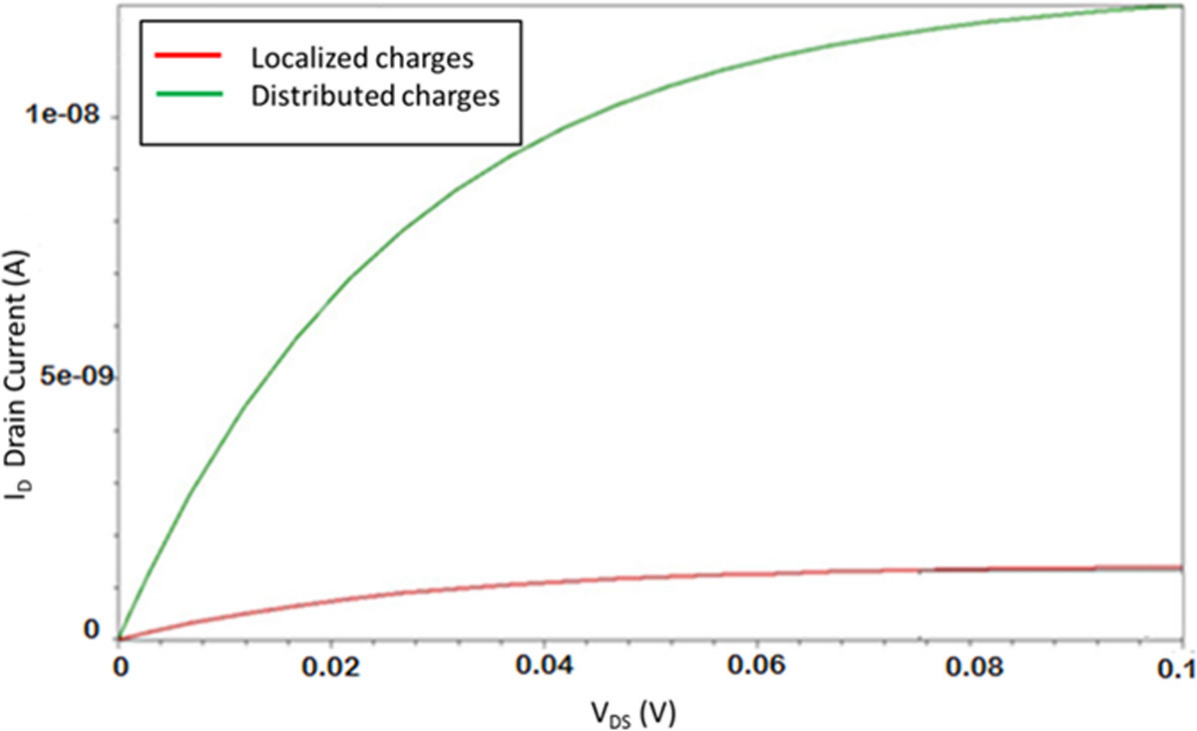
**BioFET current evaluation**. Drain current as a function of the reference electrode voltage with three active receptors: localized vs. distributed charged receptor effect on the *I_D _*current.

As mentioned before, the distribution of the charge has a strong effect on the channel modulation. For instance, if we consider three receptors *on*, localized near the source region or randomly distributed along the channel region, significantly different currents have been calculated, namely a marked increase of the current is experienced (more than one order of magnitude) when the turned on charges are more distributed along the bind sites (Figure 9 and Figure 10). These two cases have been considered as emblematic among a really huge number of simulated situations. While it is questionable if a real charge distribution can follow e.g. the situation depicted in the upper part of Figure 9, this could illustrate the capabilities of the tool, e.g. a refined analysis of different spatially localized analytes, at the same time allowing for comprehensive electric analysis of the whole device.

**Figure 11:**
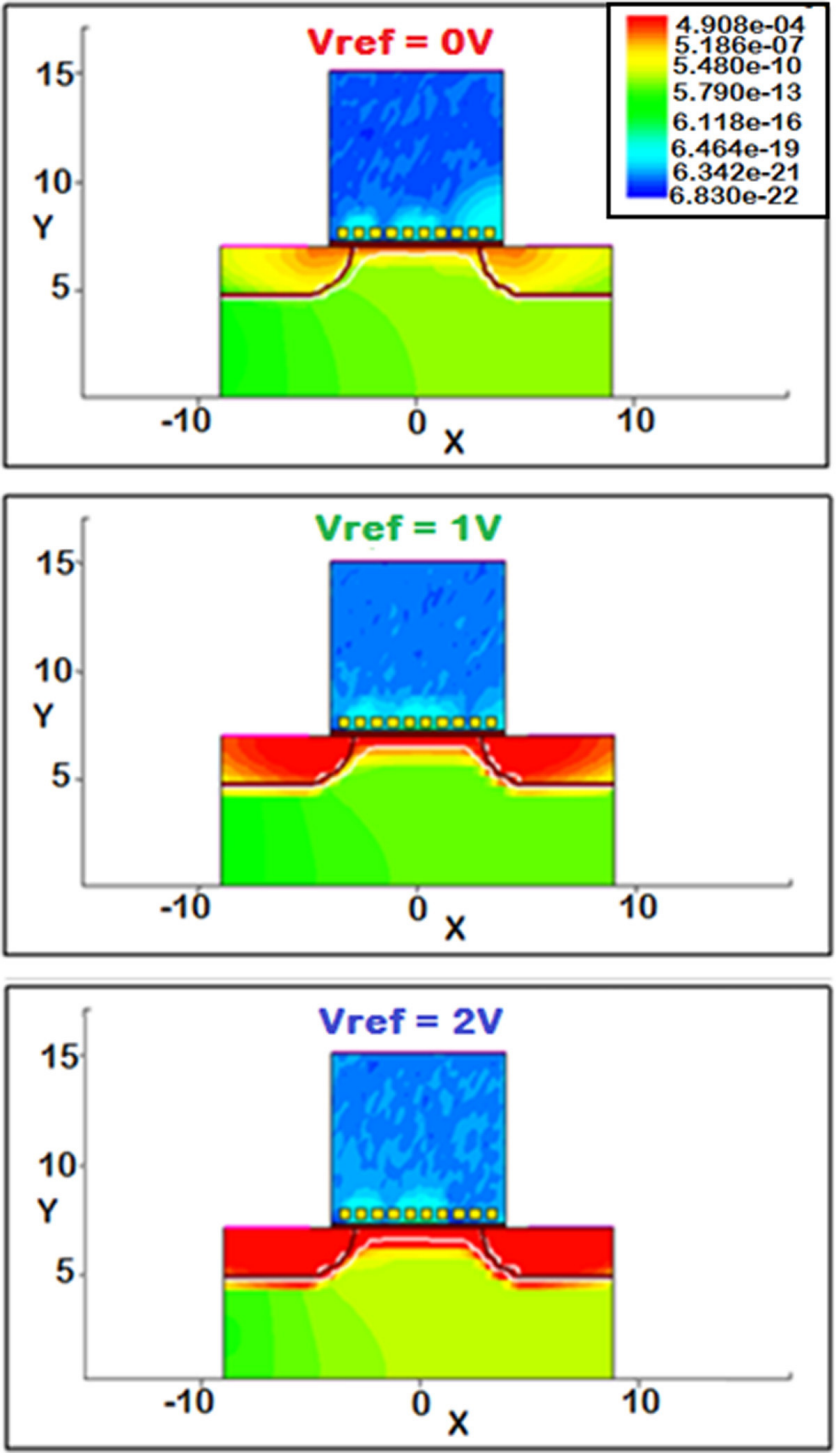
**BioFET channel modulation: voltage reference effects**. Electron current densities at different reference electrode voltages.

**Figure 12:**
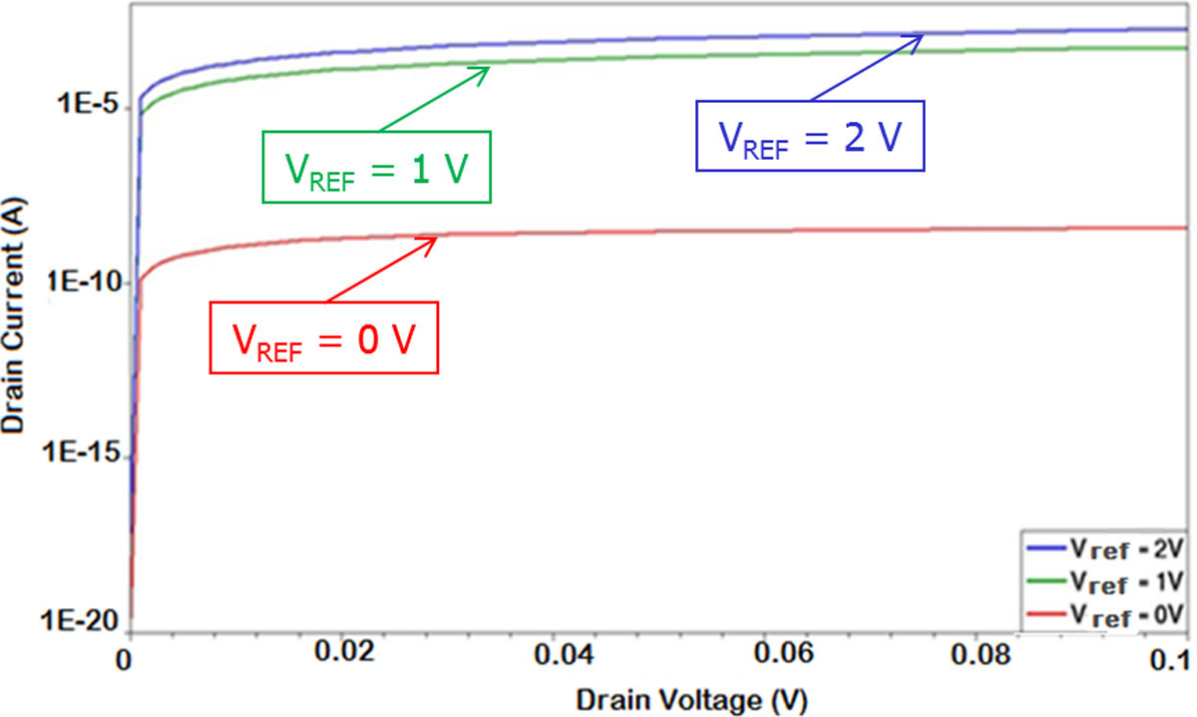
**BioFET current at different reference electrode voltages**. I_D _as a function of V_DS _at different V_REF_.

A significant effect on the overall current is related to the "biasing" of the electrolyte solution, e.g. to the reference electrode voltage. Actually, when the equivalent transistor switches in the conduction region (with respect to the sub-threshold regime) a marked increase of the drain current is obtained, as expected. This is of particular interest when setting the operating bias point of a real device (Figure 11 and Figure 12).

**Figure 13:**
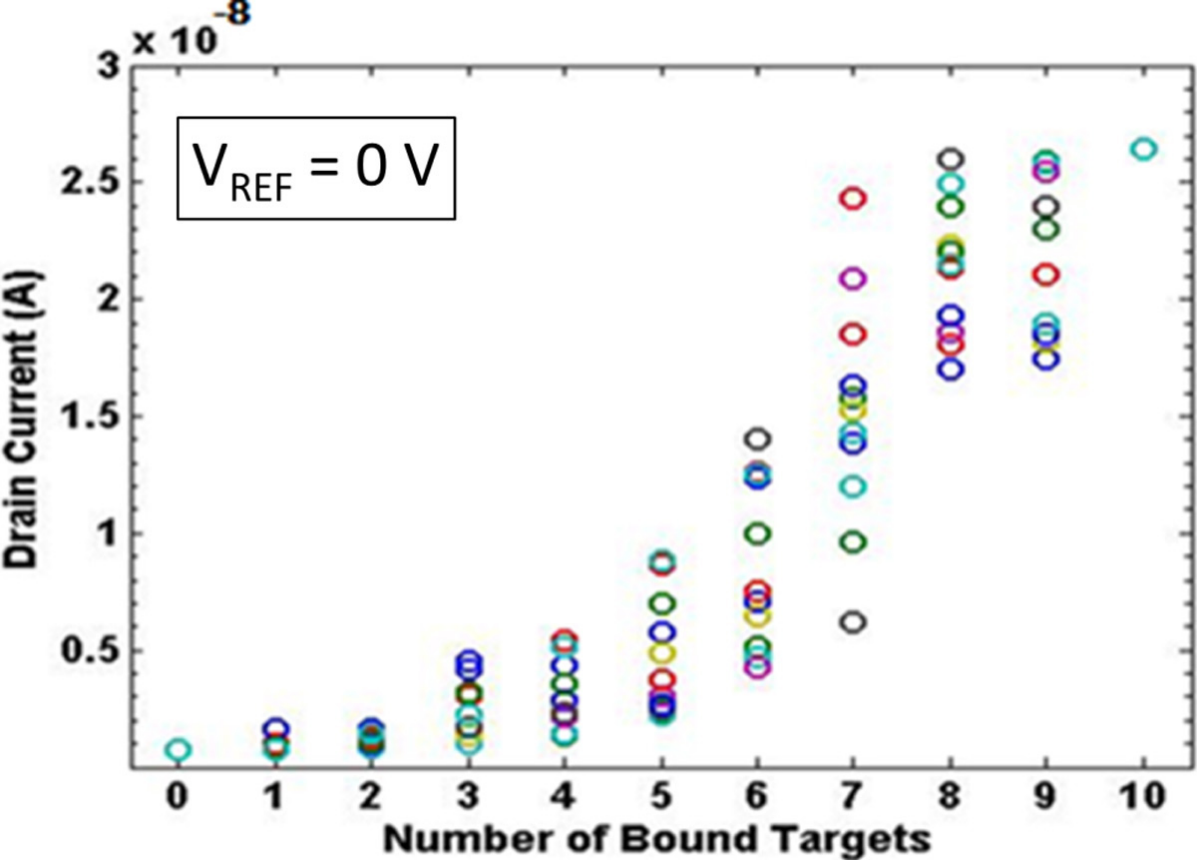
**Drain current as a function of the number of charged receptors - 0 V**. Drain current as a function of the number of charged receptors: coloured circles in each column (e.g. for the same number of charged receptors) correspond to different random position combinations (*V_DS _*= 100*mV, V_REF _*= 0V).

An overall effort aiming at summarizing the current modulation effects of an increasing number of localized charges is shown in Figure 13. It should be noted that the order of magnitude of the calculated current for the structure at hand are in agreement with literature data (simulated and measured) related to similar ISFET structures [9].

As a general comment, the relationship between current modulation and target number is not linear at all. Actually, the position of the targets has a strong effect: for instance, the localization of few targets turned on can result in a greater current modulation with respect to even a bigger number of grouped active targets. In general, charges localized near the source and drain region (C1 and C10 in the example at hand) are less effective, since the modulation of the channel is mostly affected by the influence of the lateral diffusion of source and drain region implants. Moreover, charges localized near the source (C2, C3) are more efficient in current modulation with respect to charges localized near the drain (C8, C9).

**Figure 14:**
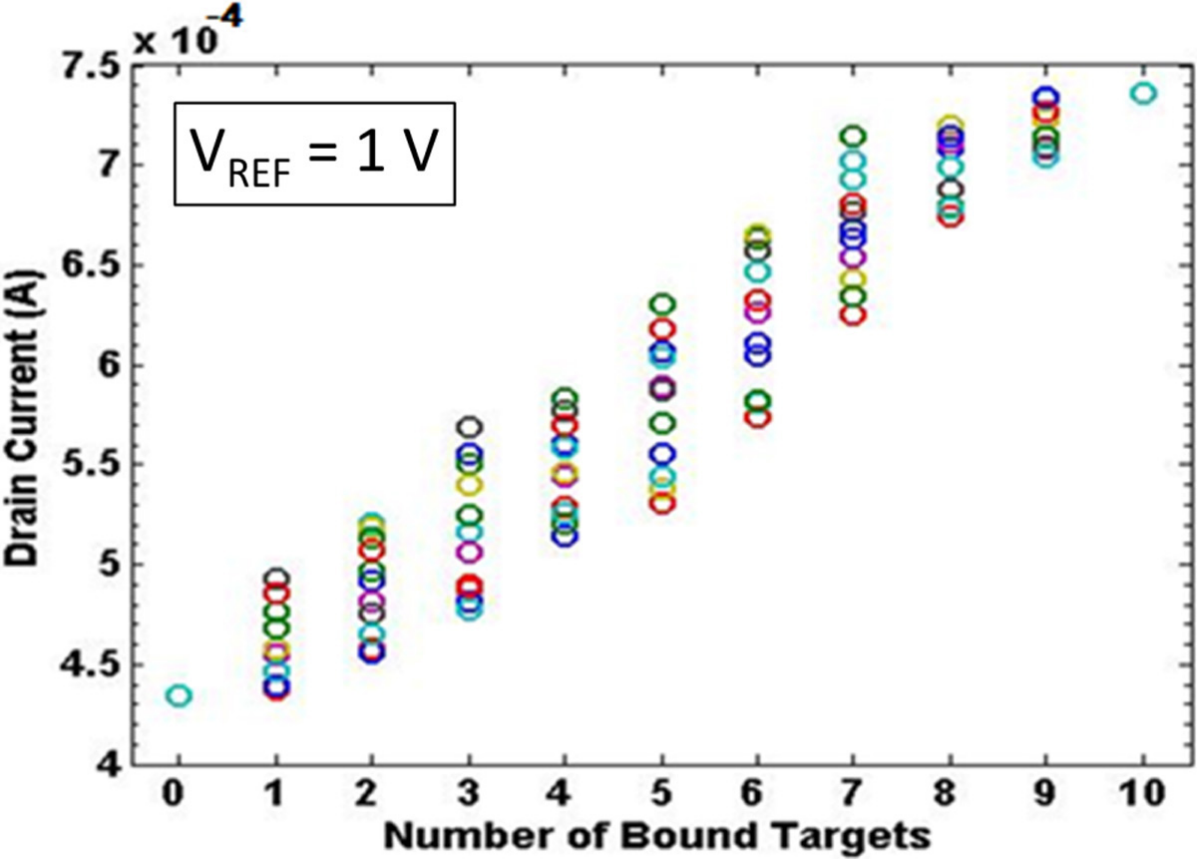
**Drain current as a function of the number of charged receptors - 1 V**. Drain current as a function of the number of charged receptors: coloured circles in each column (e.g. for the same number of charged receptors) correspond to different random position combinations(*V_DS _*= 100*mV, V_REF _*= 1V).

A further significant effect on the electrical current values is due to the reference electrode voltage. If we increase the reference voltage, a marked increase of the current (around four orders of magnitude) has been calculated. This is due to the different conducting region of the equivalent FET transistor. Even if is not straightforward to determine its threshold voltage, when the *V_REF _*= 0*V *the transistor is in the sub-threshold region, namely in a very low current regime. On the other hand, when the *V_REF _*= 1V the transistor goes on, and a much greater current flows between source and drain even when a small *V_DS _*voltage is applied. In both cases the conductive channel modulation effect is visible; a smaller modulation ratio (namely, the ratio $I_{D}^{max}/I_{D}^{min}$ between the maximum current $I_{D}^{max}$ when all receptors are on with respect to the minimum current $I_{D}^{min}$ when no charge is applied) has been obtained when *V_REF _*= 1V (Figure 14), but the bigger values of the current are a definite advantage, for instance allowing for a much simplified real experimental measurement setup. The same behaviour is further enhanced if a greater *V_REF _*is used, e.g. *V_REF _*= 2V.

## Discussion

The adopted simulation methodology (ISFET and electrolyte simulation with a commercial TCAD tool) aims at devising innovative, large BioFET sensor for neuronal activity monitoring. We adopted a simulation scheme which has been used for the analysis of "conventional" biosensors such as Silicon NanoWires (SiNW) [8] on a larger scale. The goal was to check the suitability of the approach to the study of different structures in different operating conditions. The simulated domain has been therefore suitably tailored. This allowed the comparison between the simulated results (e.g. pH sensitivity, calculated currents) with literature data [9]. Once assessed the parameters of the device and the simulation methodologies, a "segmented" ISFET structure has been devices. This device would be much bigger (hundreds of micro-meters) with respect to SiNW FET. However, in order to retain sufficient spatial resolution for the cell activity monitoring, interdigitated FET structure could be proposed. Organic FETs have been proposed as well, however with intrinsic limitation in spatial resolution [17]. Within this framework, the simulation of the structure sketched in Figure 4 has been carried out. The obtained simulation results foster the application of this "segmented" ISFET on a large scale. Actually, its sensitivity is in agreement with simulation findings obtained in [8], even for small localized charges at bigger distance from the conductive channel. Moreover, some interesting indications have been obtained, e.g. the adoption of a higher biasing reference voltage for the electrolyte solution (e.g. greater than 1 Volt) will allow much easier current measurements, at the same time retaining a good sensitivity.

The final goal of this study would be the proof of concept of the feasibility of an integrated high-precision multi-channel system capable of stimulating neural activity while recording very low-voltage responses as low as tens of microvolts. A lumped-element prototype of this system is currently under study within the framework of an international collaboration including the authors of this paper [18].

## Conclusions

In this work we presented a numerical simulation approach to the study of Ion-Sensitive Field Effect Transistor (ISFET) structures for biosensing devices (BioFETS) using the Synopsys Sentaurus Technology Computer-Aided Design (TCAD) tools. In particular, we concentrate on the analysis of the field effect on the conduction channel of a general BioFET structure that leads to the current modulation due to the fixed charges induced by immobilization of target biomolecules in an electrolyte environment. The channel modulation effect induced by irregular, locally distributed charges can be deeply investigated by means of device-level numerical simulation, as well as the effects of different electrolyte concentrations (*pH*) on the device sensitivity.

In this way a powerful framework for the design and optimization of biosensor can be devised, thus reducing technology development time and cost. The main finding of the analysis of a general reference BioFET shows that there is no linear relationship between the number of charges and the current modulation, but there is a strong position dependent effect: targets localized near the source region are most effective with respect to targets localized near the drain region, and in general even randomly distributed targets are more efficient with respect to locally grouped targets on the current modulation. The effect of the *V_DS _*drain source voltage on the sensitivity of the device, as well as the effect of the different polarization of the electrolyte reference voltage (*V_REF_*) can be studied in detail. In particular, for the device at hand, a small positive biasing of the electrolyte solution, providing that the transistor goes on, will result in a greater enhancement of the current levels, still retaining a good sensitivity but greatly simplifying the operations of a real device.

## Competing interests

The authors declare that they have no competing interests.

## Authors' contributions

DP conceived and designed the study, coordinated the simulation activities and mainly contributed in the data analysis and in the writing of the manuscript. AM and KK carried out the model parameterization and simulation studies and contributed in the data analysis. AS contributed in drafting the manuscript and revising it critically for important intellectual content. All authors read and approved the final manuscript.

## Declarations

Publication of this article was supported by Italian MIUR research project PRIN 2010/2011 ARTEMIDE (Autonomous Real Time Embedded Multi-analyte Integrated Detection Environment).

This article has been published as part of *BioMedical Engineering OnLine *Volume 14 Supplement 2, 2015: Select articles from the 2nd International Work-Conference on Bioinformatics and Biomedical Engineering (IWBBIO 2014). The full contents of the supplement are available online at http://www.biomedical-engineering-online.com/supplements/14/S2.
